# Design of Ultra-Compact and High-Efficiency Waveguide Bends on Lithium Niobate Thin Films

**DOI:** 10.3390/nano16161004

**Published:** 2026-08-15

**Authors:** Yi-Wen Wang, Zhi-Chen Wei, Xiao-Dong Wen, Tian-Xue Ma

**Affiliations:** 1 School of Mathematics and Physics, Lanzhou Jiaotong University, Lanzhou 730070, China; zhichenweilzjtu@163.com (Z.-C.W.); wenxiaodong@lzjtu.edu.cn (X.-D.W.); 2 Gansu Center for Fundamental Research in Complex Systems Analysis and Control, Lanzhou Jiaotong University, Lanzhou 730070, China; 3 Department of Mechanics, School of Physical Science and Engineering, Beijing Jiaotong University, Beijing 100044, China

**Keywords:** integrated optics, lithium niobate thin film, waveguide bends

## Abstract

This paper systematically investigates the structural design and numerical characterization of ultra-compact low-loss waveguide bends on lithium niobate-on-insulator (LNOI) thin films via 3D-FDTD simulations. Six 90° bend architectures are analyzed to unravel transmission behaviors and intrinsic loss mechanisms, including three smooth bend optimizations: straight–bend lateral offset, local width tapering, and Euler–circular hybrid curvature modulation, alongside resonant-cavity and corner-mirror L-shaped bends. All structures achieve evident loss reduction within proper parameter windows. At *R*_eff_ = 5 μm, optimized smooth bends reach a minimum loss of 0.046 dB/90°, outperforming standard circular bends, while the double-corner-mirror bend exhibits the lowest loss of 0.467 dB/90° among right-angle configurations. Pairwise parametric scans disclose competitive effects among diverse loss-mitigation pathways, demonstrating that simultaneous use of two optimization strategies fails to cut extra loss at equal device dimensions. Broadband and fabrication tolerance simulations verify flat spectral response across the telecom C band; smooth curved bends possess strong robustness against inclined sidewalls, and etch depth acts as the dominant factor governing device loss. This work delivers systematic parametric guidelines for the design and optimization of miniaturized LNOI routing waveguides for photonic interconnects.

## 1. Introduction

Lithium niobate (LN) possesses excellent electro-optic, nonlinear optical, acousto-optic, piezoelectric, and pyroelectric properties, rendering it an important functional material in integrated photonics [1,2,3,4]. With the advancement of fabrication technologies such as ion implantation and wafer bonding, the lithium niobate-on-insulator (LNOI) platform composed of single-crystal LN thin films and low-refractive-index SiO_2_ achieves a high refractive index contrast and strong optical confinement, making LNOI an important platform for high-density photonic integrated circuits [5,6,7,8]. Beyond these general platform advantages, the thin-film geometry enables subwavelength optical confinement, while the non-centrosymmetric LN crystal provides the Pockels effect and second-order nonlinear interactions required for high-speed electro-optic modulation and nonlinear frequency conversion [4,9]. Optimized dry etching has also demonstrated low-loss, strongly confined monolithic LNOI waveguides, supporting compact integrated devices with tight bending radii [9]. Devices based on LNOI, including modulators [10,11,12,13], detectors [14,15], conversion devices [16,17], and couplers [18,19], have been extensively studied. Recent demonstrations of packaged broadband TFLN electro-optic modulators, low-loss fiber–chip interfaces, monolithically integrated RF-photonic receivers, and compact inverse-designed crossings further show that LNOI photonics is moving from individual devices toward denser system-level circuits [20,21,22,23]. This system-level progression highlights the need for compact, low-loss passive routing elements because bend loss can otherwise limit the practical density of TFLN circuits that combine active electro-optic functions with passive interconnects [24].

Waveguide bends represent essential building blocks in the implementation of photonic components, including resonators, interferometers, delay lines, and power splitters/combiners [25,26,27,28,29]. To minimize the chip footprint, practical photonic layouts typically demand a minimized bending radius; nevertheless, a reduced bend radius intensifies outward radiation leakage and induces a more severe mode discontinuity at the straight–bend junction, resulting in significantly elevated insertion loss. In LNOI, this compactness-loss trade-off is particularly important for small-radius 90° bends, where the strongly confined mode must be redirected while suppressing sidewall-scattering-related loss and output mode mismatch [9]. Practical dry-etched LNOI waveguides may also deviate from ideal rectangular cross-sections; in particular, slanted sidewalls can induce polarization-related mode hybridization and crosstalk in representative components such as tapers and bends [30]. Furthermore, additional leakage and coupling losses may emerge depending on the specific waveguide configuration and optical polarization state [31,32,33,34]. Therefore, achieving effective suppression of bending loss while maintaining structural compactness remains an important design issue for high-density photonic integration on LNOI platforms.

Recent advances in compact LNOI/TFLN bends leverage hybrid integration, inverse design and Bézier/Euler curvature shaping to balance device compactness and bending loss [35,36,37,38,39,40]. Hybrid Si-LN waveguides achieve simulated losses below 0.001 dB/90° for radii >3 μm [35], while TFLN–chalcogenide free-form bends with a 10 μm effective radius exhibit measured losses of ~0.25 dB per bend [36]. Directly etched LNOI bends (6–10 μm radius), including inverse-designed, Bézier and Euler configurations, have been experimentally verified, with typical single-bend losses ranging from 0.4 dB to 1.2 dB [37,38,39,40]. Micrometer-scale compact bends represent the mainstream research target, yet fabricated devices generally incur loss above 0.2 dB. Enlarging the radius to 30–100 μm cuts loss to ~0.05 dB/90° at the cost of a substantially enlarged chip footprint [41,42].

To address the tradeoff between footprint and bending loss, interface geometric tuning that relies on lateral offset and width adjustment has been validated for mode matching at straight–straight junctions in low-index-contrast SiN platforms [43]. Such methods suppress interface mode mismatch loss in straight waveguide layouts without cumulative radiative leakage, yet they are only functional for linear waveguides and fail to eliminate curvature-induced radiative loss intrinsic to LNOI bends. The high index contrast of LNOI enables ultra-compact bending, but severe mode distortion and persistent radiation leakage degrade the transmission of tight bends fundamentally. Owing to distinct material properties and loss mechanisms, SiN straight-waveguide optimization rules cannot be directly transferred to LNOI bend designs. Unlike prior single-parameter interface optimization limited to SiN straight waveguides, this work develops three geometric schemes targeting diverse bending loss origins and systematically evaluates their loss mitigation performance for both smooth curved and right-angle bends under unified simulation conditions.

To address the trade-off between compactness and transmission loss in small-radius LNOI waveguide bends, we systematically evaluate six 90° waveguide bends via three-dimensional finite-difference time-domain (3D-FDTD) simulations under consistent material, cross-sectional, and numerical settings. For smooth bends, lateral offset compensates for modal-centroid displacement between straight and curved waveguides; local width reduction tailors modal confinement and radiation loss; and a Euler–circular hybrid curve mitigates junction discontinuities through continuous curvature variation, while for L-shaped bends, resonant-cavity, single-corner-mirror, and double-corner-mirror designs are compared as different field-redirecting schemes. Based on single-parameter optimizations, pairwise scans of the Euler proportion *η*, lateral offset Δ*x*, and local width reduction Δ*w* reveal coupling among the smooth bend strategies, and the optimized structures are further characterized by 1260–1625 nm wavelength sweeps, fabrication-tolerance analysis, and validation against experimentally reported LNOI bends of similar size, assessing their spectral response, fabrication robustness, and loss realism, with numerical reliability verified through mesh refinement, PML boundary tests, and cross-checking with an eigenmode expansion method. By linking bending loss to mode matching, radiation leakage, and field reconstruction, this work clarifies the underlying mechanisms, applicable regions, and design trade-offs of the various strategies.

## 2. Simulation Study of Waveguide Bends

**Figure 1:**
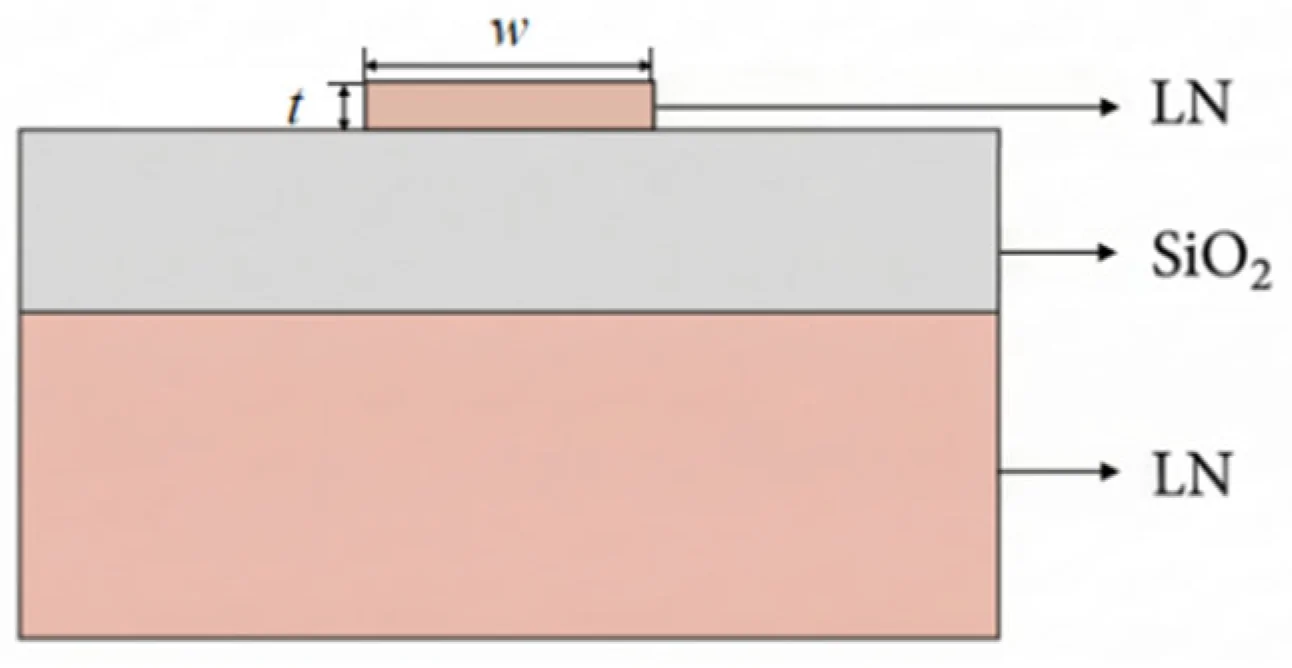
Cross-section of the LN bent waveguide.

All waveguides are fabricated on z-cut LN thin films, with an operating wavelength of 1.55 μm, a waveguide thickness *t* = 0.5 μm, and a waveguide width *w* = 1 μm. The cross-sectional structure of the waveguide is illustrated in Figure 1. Numerical simulations are performed using the FDTD method. Perfectly matched layer (PML) boundary conditions are applied to all simulation boundaries to eliminate the influence of boundary reflections on the calculated results. Power monitors are placed at the input and output ends of the curved structure to obtain the transmittance, based on which the 90° bending loss is calculated. The transmittance *T* is defined as the ratio of the output optical power *P*_out_ to the input optical power *P*_in_, $T=P_{\mathrm{out}}/P_{\mathrm{in}}$. The bending loss, expressed in dB/90°, is given by:

(1) $$\mathrm{Loss}=-10\log_{10}(T)$$

In the subsequent comparative analysis, to ensure the comparability of the results, the cross-sectional dimensions, material parameters, and simulation conditions of the waveguides are kept consistent. Simulations are conducted for equivalent bending radii *R* = 2, 3, 4, and 5 μm, respectively.

To verify the numerical reliability of the reported bend losses, we performed comprehensive mesh-convergence tests, PML-distance checks, and independent EME-model validation. All losses were calculated using the same definition, *L* = −10 log_10_ *T* = −10 log_10_ |*S*_21_|^2^, where *T* = |*S*_21_|^2^ is the normalized fundamental-mode transmittance. A one-factor-at-a-time procedure was used for the convergence tests: except for the in-plane mesh step or the distance between the waveguide core and the PML under examination, the device geometry, material parameters, excitation mode, input and output monitors, out-of-plane mesh, PML type and number of layers, simulation time, and auto-shutoff criterion were held fixed. Detailed numerical simulation settings, convergence tests, and independent-model validation are provided in Appendix A.

**Figure 2:**
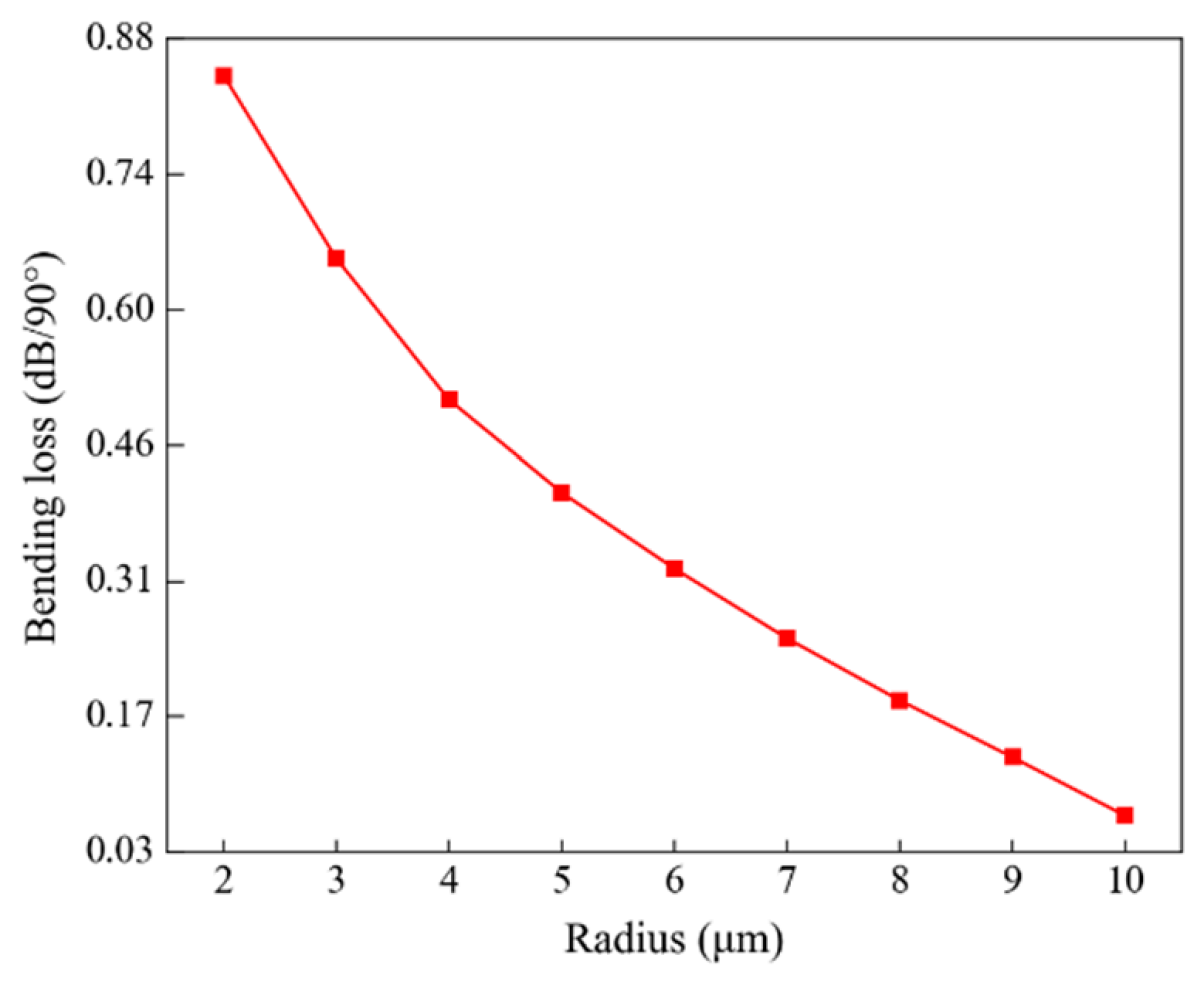
Relationship between bending radius and bending loss in conventional waveguide bends.

Figure 2 shows the bending loss of conventional bent waveguides as a function of the bending radius. The loss decreases monotonically with increasing bending radius. A significant variation is observed when the bending radius falls below 5 μm. Specifically, reducing the bending radius from 5 μm to 2 μm results in an additional loss of 0.63 dB/90°.

### 2.1. Lateral Offset

**Figure 3:**
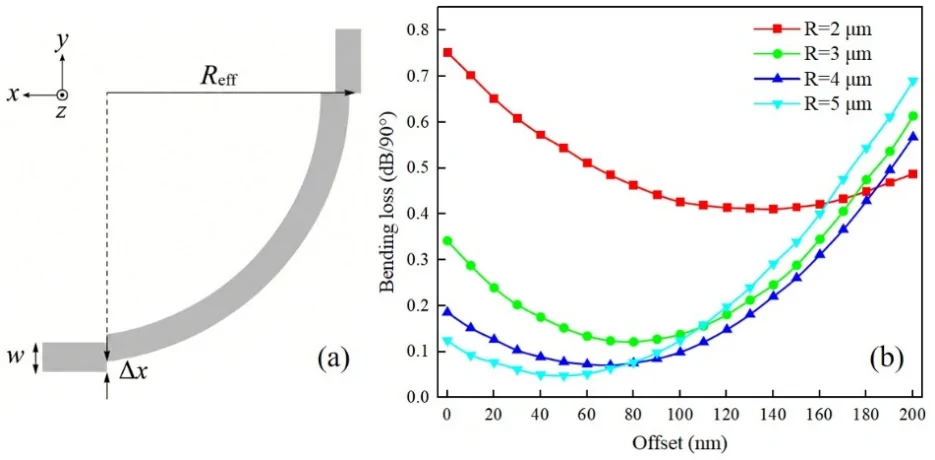
(**a**) Schematic of the lateral offset in a waveguide bend; (**b**) relationship between lateral offset Δ*x* and bending loss for different bending radii.

To reduce the transition loss at the straight–bend interface, a lateral offset Δ*x* is incorporated into the waveguide bend. As shown in Figure 3a, the structure consists of input and output straight waveguides connected by a bend, where *w* denotes the waveguide width, *R*_eff_ represents the equivalent bending radius, and Δ*x* indicates the relative lateral offset at the straight–bend interface. In this model, a single variable control method is adopted. Only the lateral offset Δ*x* at the straight–bend interface is adjusted to improve the spatial overlap of the guided modes on both sides, thereby reducing the loss caused by mode mismatch.

Figure 3b presents simulation results for transverse offsets Δ*x* ranging from 0 to 200 nm. For bending radii of 2, 3, 4, and 5 μm, the bending loss first decreases and then increases with increasing offset. The minimum losses are 0.41, 0.12, 0.07, and 0.05 dB/90°, achieved at optimal offsets of 140, 80, 70, and 50 nm, respectively. Compared with the zero-offset case, the loss reductions are 0.34, 0.22, 0.12, and 0.07 dB/90°, respectively. These results demonstrate that an appropriate transverse offset at the straight–bend interface effectively reduces bending loss, with a smaller bending radius requiring a larger optimal offset. The bending loss arises from two main sources: (1) transition loss due to curvature discontinuity and mode mismatch at the straight–bend junction; (2) radiation loss caused by the outward shift of the guided mode and subsequent leakage into the cladding along the bent section. Although the straight and bent waveguides share identical geometric parameters at the interface, their modal characteristics differ, leading to mode mismatch and additional transition loss [44,45]. Bending breaks the symmetry of the effective refractive index distribution, pushing the mode field outward. This effect intensifies as the bending radius decreases, exacerbating both mode mismatch and radiation loss [46]. Introducing a proper transverse offset compensates for the mode-field center deviation induced by the outward mode shift in the bend, thereby enhancing mode overlap between the straight and bent waveguides and reducing propagation loss. Introducing a proper transverse offset compensates for the mode-field center deviation caused by bending, enhancing mode overlap and reducing transition loss. This method mainly suppresses junction-induced transition loss but cannot reduce distributed radiation loss along the curved waveguide. Thus, loss improvement is more significant at smaller radii where interface mismatch dominates. As the radius increases to 3–5 μm, radiation loss gradually saturates, leading to similar minimum loss levels.

**Figure 4:**
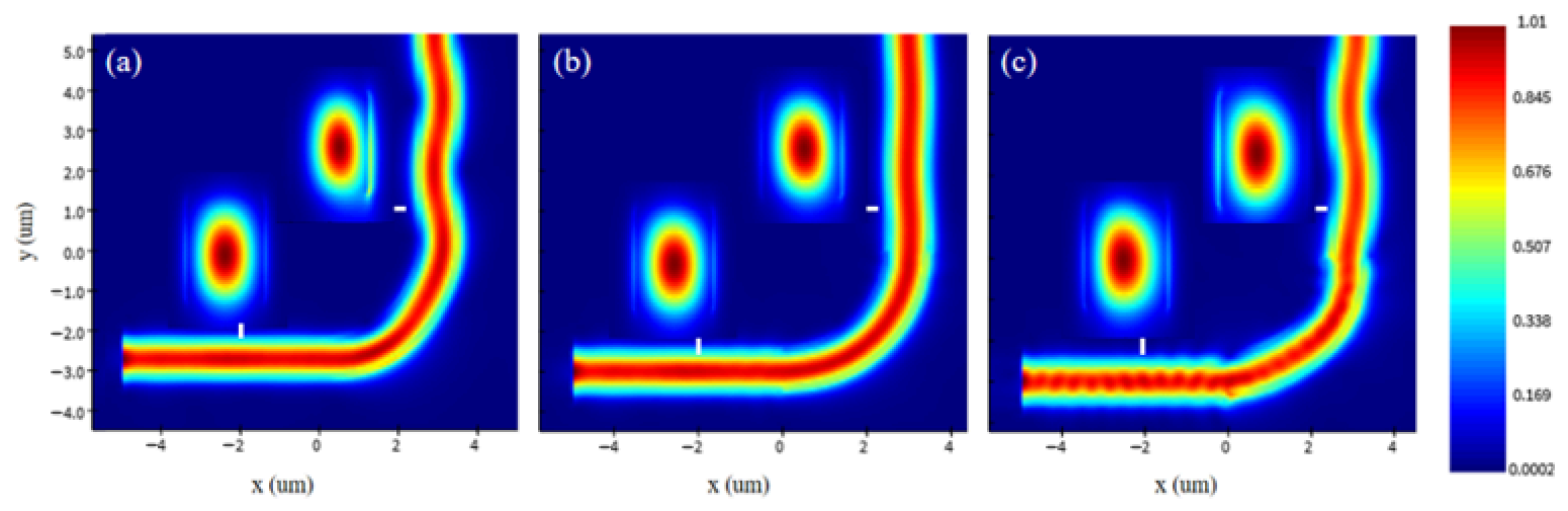
E-field distributions for different Δ*x* at a bending radius of 3 μm: (**a**) Δ*x* = 0 nm; (**b**) Δ*x* = 80 nm; (**c**) Δ*x* = 200 nm.

Figure 4 presents simulated electric-field (E-field) distributions under different transverse offsets. The embedded insets show transverse E-field profiles extracted at the input cross-section (*x* = −2 μm) and output cross-section (*y* = 1 μm). At Δ*x* = 0 nm (Figure 4a), the field exhibits noticeable distortion at the straight–bend junction and a broad energy spread on the outer bend side, indicating severe mode mismatch and substantial radiation leakage. In contrast, at Δ*x* = 80 nm (Figure 4b), the field transition becomes smoother, the energy distribution within the bend is more continuous, and the output mode field is better confined, confirming that an appropriate offset improves mode overlap and suppresses bending loss. However, at Δ*x* = 200 nm (Figure 4c), pronounced field fluctuations emerge in the input straight waveguide and near the junction, suggesting that overcompensation introduces mode mismatch and scattering loss, leading to a renewed increase in propagation loss. 

### 2.2. Reducing the Width of the Bent Section

**Figure 5:**
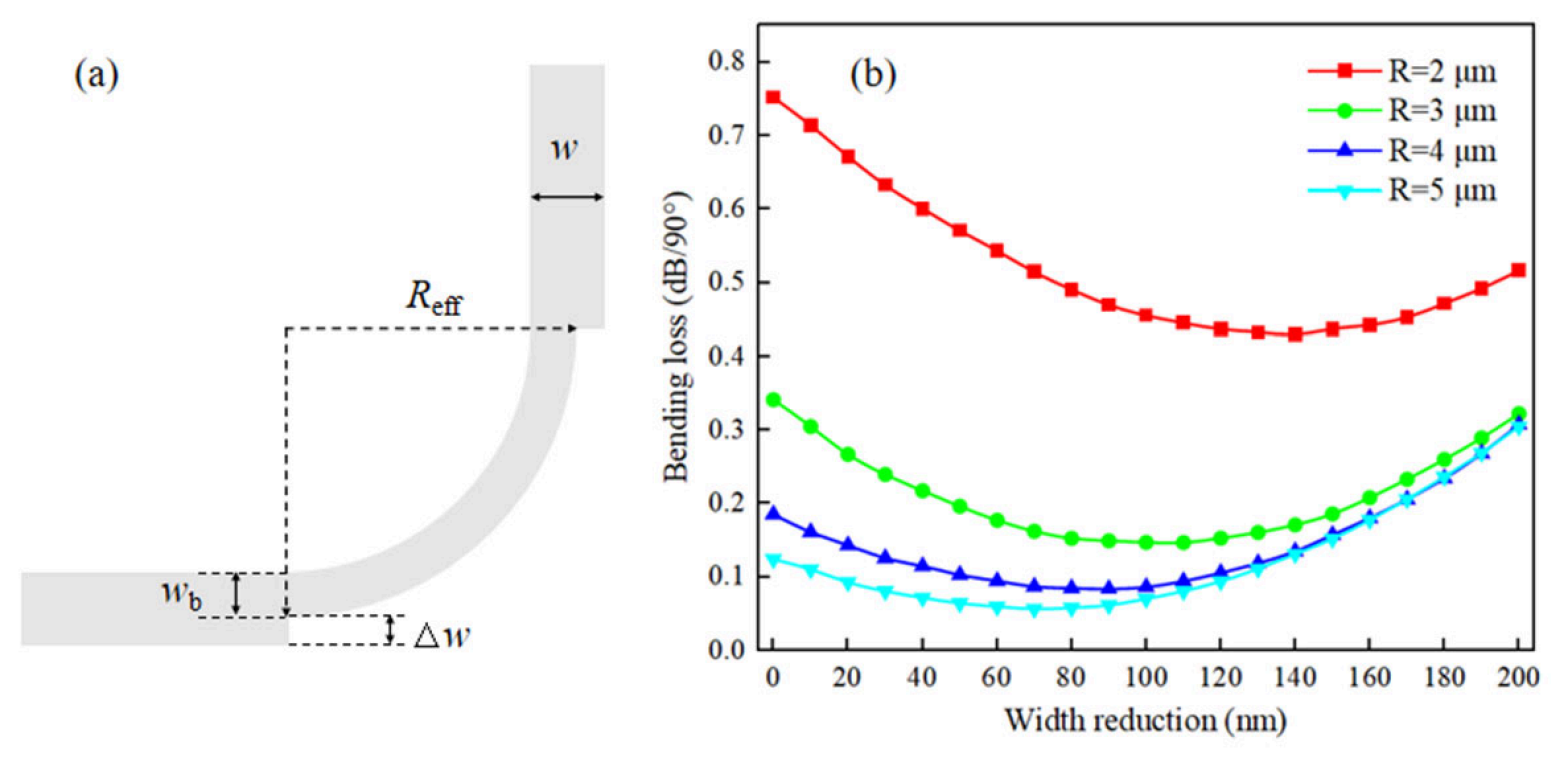
(**a**) Schematic diagram of local width reduction in a waveguide bend; (**b**) relationship between local width reduction and bending loss in bent waveguides for different bending radii.

To investigate the effect of local width reduction on the transmission characteristics of the waveguide bend, a structural model incorporating width modulation exclusively in the bent section was established, as illustrated in Figure 5a. The widths of both the input and output straight waveguides are maintained at *w*, while only the width of the bent section is reduced to *w*_b_ = *w* − Δ*w*, where *R*_eff_ denotes the effective bending radius and Δ*w* represents the local width reduction of the bent section relative to the straight waveguide. In this model, the single-variable method is adopted, varying solely the width of the bent section. The mode-field distribution and energy confinement within the bent region are tailored by adjusting its transverse dimension, thereby investigating the effect of local width reduction on the bending loss.

Figure 5b shows the bending loss as a function of local width reduction in the bent section, with the straight waveguide width fixed at *w* = 1 μm and *w*_b_ = *w* − Δ*w* (Δ*w* = 10–200 nm). For effective radii *R*_eff_ = 2, 3, 4, and 5 μm, the bending loss first decreases and then gradually increases with increasing Δ*w*. The minimum losses are 0.43, 0.15, 0.08, and 0.05 dB/90°, achieved at optimal width reductions of 140, 100, 90, and 70 nm, respectively. Compared with conventional structures without width reduction, the loss reductions are 0.32, 0.19, 0.11, and 0.06 dB/90°, respectively. These results indicate that moderately reducing the width of the waveguide bend can lower bending loss. The total bending loss is primarily determined by mode mismatch loss at the straight–bend junction and radiation loss inherent to the curved section. Bending shifts the guided mode toward the outer side of the bend, strengthens the evanescent field on that side, and exacerbates both interface mode mismatch and radiation leakage. These effects become more pronounced as the bending radius decreases [47]. Local width tapering mainly suppresses radiation loss along the curved waveguide. Narrowing the waveguide strengthens lateral optical confinement and pulls the mode centroid back toward the waveguide axis, reducing evanescent leakage. Excessive tapering, however, introduces additional interface mode mismatch loss. Bending loss optimization thus requires a trade-off between radiation loss and transition loss, yielding an optimal width-reduction window for each bending radius.

**Figure 6:**
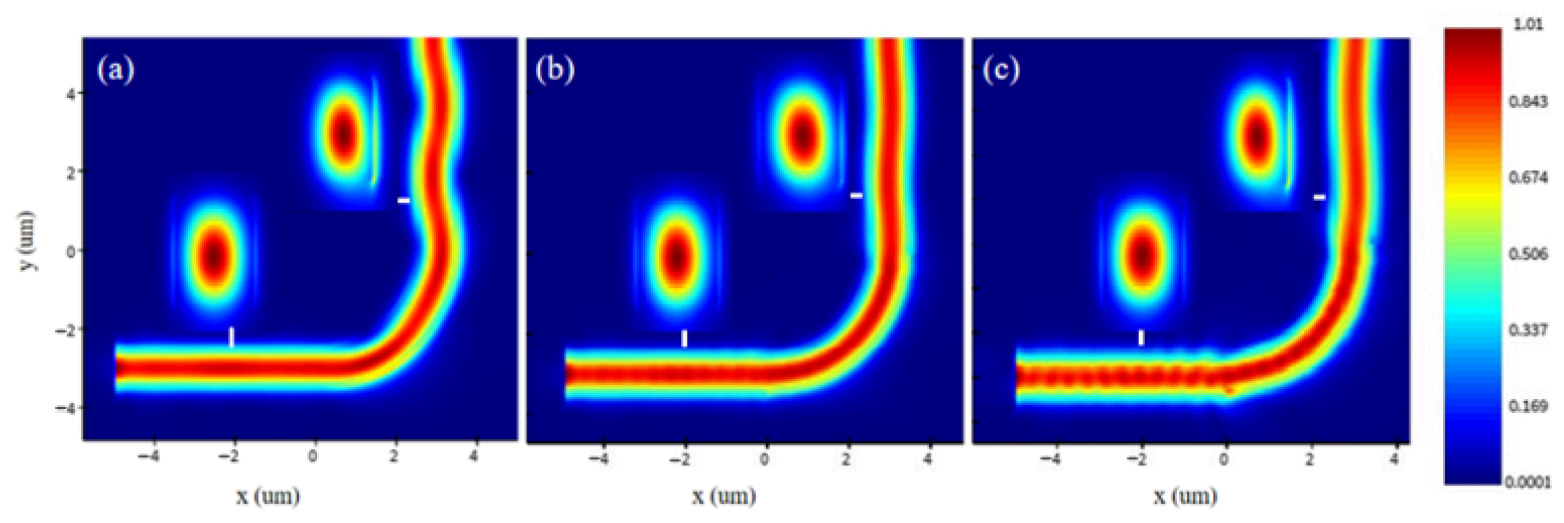
E-field distributions for different Δ*w* at a bending radius of 3 μm: (**a**) Δ*w* = 0 nm; (**b**) Δ*w* = 100 nm; (**c**) Δ*w* = 200 nm.

As shown in Figure 6, the embedded insets show transverse E-field profiles extracted at the input cross-section (*x* = −2 μm) and output cross-section (*y* = 1 μm). The E-field exhibits noticeable outward diffusion at Δ*w* = 0 nm (Figure 6a), indicating persistent radiation leakage during bending. At Δ*w* = 100 nm (Figure 6b), the field becomes more confined within the bent region, with suppressed outer leakage and a more concentrated output mode, suggesting that a moderate width reduction enhances optical confinement and reduces transmission loss. In contrast, at Δ*w* = 200 nm (Figure 6c), pronounced field fluctuations and local perturbations appear in the straight waveguide and straight–bend transition region, reflecting increased mode mismatch and scattering due to excessive width reduction, which elevates the total loss relative to the Δ*w* = 100 nm case. These results demonstrate that while local width tapering can mitigate bending-induced radiation loss, an optimal tapering range exists beyond which the loss performance degrades. 

### 2.3. Euler–Circular Hybrid Bend

**Figure 7:**
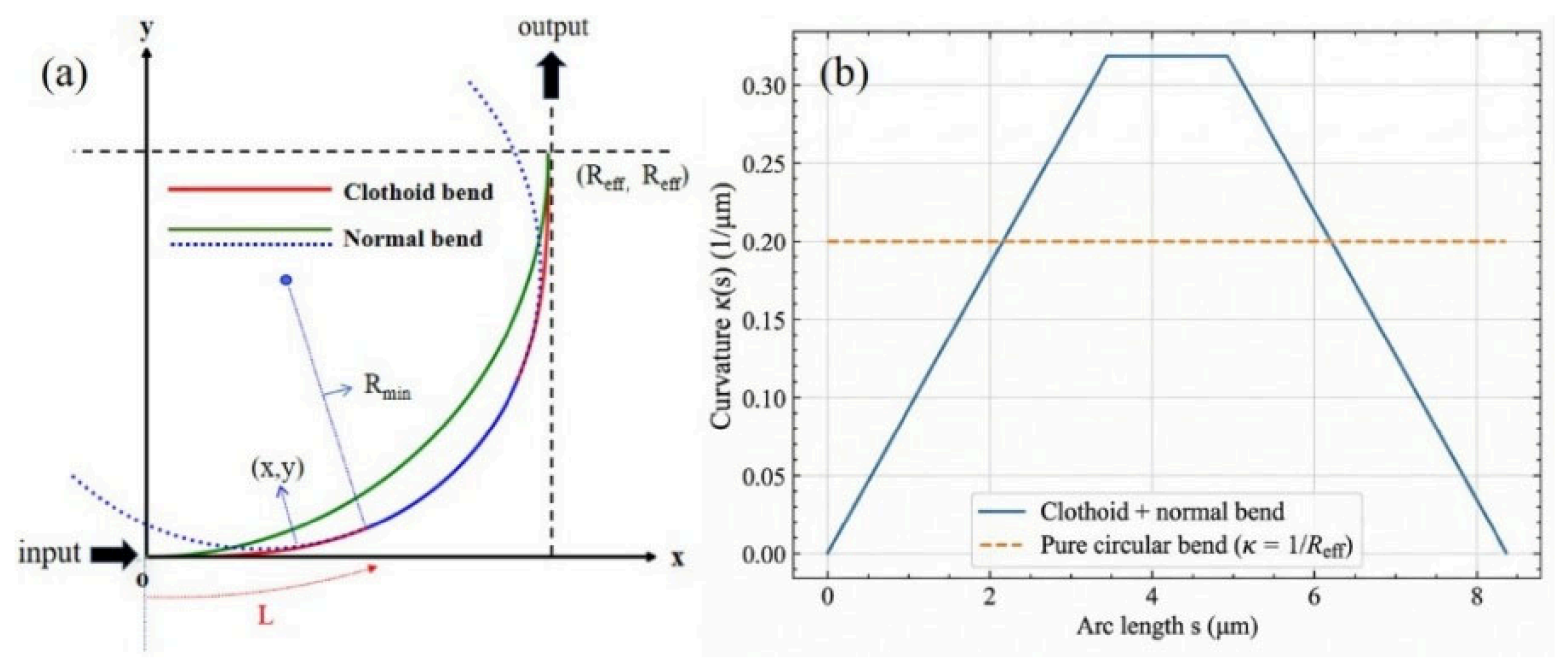
(**a**) Schematic of the Euler–circular hybrid bend structure; (**b**) comparison of the centerline curvature *κ*(*s*) distribution between the Euler–circular hybrid bend and the pure circular bend.

To mitigate the additional loss caused by abrupt curvature changes in conventional circular bends, this work adopts a hybrid bend consisting of Euler bends and a circular arc, as shown in Figure 7a. The waveguide starts from the origin, undergoes a 90° rotation along the propagation direction, and terminates at (*R*_eff_, *R*_eff_). Euler transition segments are introduced at both ends of the 90° bend, with a constant-curvature circular arc retained in the middle. The parameter *η* characterizes the proportion of the Euler bend in the overall structure. When *η* = 0, the structure degenerates into a conventional circular bend. As *η* increases, the curvature *κ*(*s*) transitions from an abrupt step to a continuous variation along the propagation direction. The key advantage of the Euler bend lies in improving the straight–bend junction condition via a smooth curvature transition, thereby reducing the additional bending loss within a certain range [48,49].

The coordinates of the Euler bend in the plane can be expressed as:

(2) $$x=A\int_{0}^{L/A}\sin\left(\frac{\theta^{2}}{2}\right)d\theta$$

(3) $$y=A\int_{0}^{L/A}\cos\left(\frac{\theta^{2}}{2}\right)d\theta$$

where *A* is the parameter of the Euler bend, which controls the overall scale of the curve, and *θ* is the parametric variable of the Euler bend. Meanwhile, the curvature radius *R* corresponding to a point (*x*, *y*) on the Euler bend and the curve length *L* satisfy the following relation:

(4) $$RL=A^{2}$$

This geometric relation is employed to achieve matching between the Euler bend segment and the intermediate circular arc segment during structural design, thereby ensuring geometric continuity of the entire 90°waveguide bend.

The proportion of the Euler bend in the entire 90° bending path is defined as:

(5) $$\eta =\frac{L_{\mathrm{Euler}}}{L_{\mathrm{total}}}$$

where $L_{\mathrm{Euler}}$ and $L_{\mathrm{total}}$ are the lengths of the Euler segment and the entire bending path, respectively. The bending loss is systematically investigated as a function of η. The equivalent footprint area is kept constant across all comparisons, ensuring that the planar layout of the Euler–circular hybrid bend matches that of conventional bends. This allows a fair comparison under identical spatial constraints, demonstrating that the proposed structure simultaneously maintains high integration density and reduces bending loss through an optimized composite curve.

Figure 7b illustrates the continuous curvature variation introduced by the Euler bend segment at the straight–bend interface. The distribution of the waveguide centerline curvature *κ*(*s*) as a function of the propagation arc length s is calculated and plotted. Taking *R*_eff_ = 5 μm as an example, the curvature of a pure circular bend is constant, given by *κ* = 1/*R*_eff_. In contrast, for the Euler–circular composite bend, the curvature increases linearly from zero at the input end, remains constant at 1/*R*_min_ over the intermediate circular arc segment, and then decreases symmetrically to zero at the output end. Without altering the endpoint occupancy (with the terminal point fixed at (*R*_eff_, *R*_eff_)), the composite structure achieves a smoother curvature transition in the straight bend region, thereby reducing mode mismatch and excess radiation loss caused by abrupt curvature changes.

**Figure 8:**
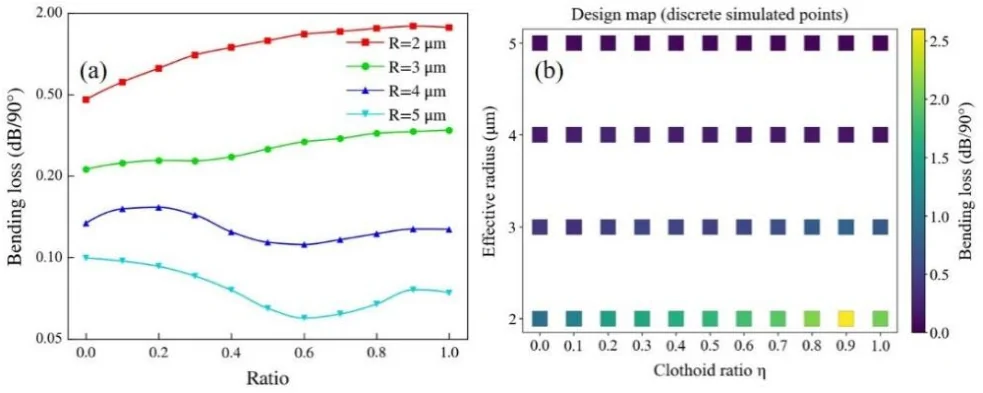
(**a**) Relationship between the ratio *η* of the Euler–circular hybrid bend structure and bending loss for different bending radii; (**b**) optimal region diagram of the Euler–circular hybrid bend structure.

Figure 8a presents the simulation results. As the curve proportion *η* increases, the bending loss exhibits distinct trends depending on the effective bending radius. For *R*_eff_ = 2 and 3 μm, the minimum losses are 0.75 dB/90° and 0.34 dB/90°, respectively, both occurring at *η* = 0 (conventional circular bend). For *R*_eff_ = 4 and 5 μm, the loss shows a pronounced non-monotonic variation with *η*, reaching a minimum *η* = 0.6. Specifically, at *R*_eff_ = 4 μm, the loss decreases to 0.15 dB/90° at *η* = 0.6, a reduction of 0.04 dB/90° compared with 0.19 dB/90° at *η* = 0. At *R*_eff_ = 5 μm, the loss drops to 0.06 dB/90° at *η* = 0.6, a reduction of 0.06 dB/90° from 0.12 dB/90° at *η* = 0. These results indicate that the Euler–circular hybrid bend effectively reduces bending loss under relatively large equivalent radii. Figure 8b also provides an optimal region diagram, offering a more intuitive illustration of the relationship between bending loss, *η*, and *R*_eff_.

According to the bend-loss mechanism, the limited benefit of the Euler segment at very small radii can be understood from the standard loss-decomposition picture, in which the total loss consists of a transition component generated at regions of rapid curvature variation and a radiative bend-mode component generated in high-curvature regions [48]. Increasing the Euler proportion *η* smooths the straight–bend transition and reduces transition loss. However, because the hybrid bend is designed under a fixed footprint, a larger *η* compresses the central circular arc and reduces the minimum local radius of curvature *R*_min_ required to complete the 90° turn within the same area. The radiative component is governed by *R*_min_ and increases sharply as *R*_min_ decreases. Consequently, the total bending loss is determined by a competition between transition-loss reduction and radiation-loss enhancement. For *R*_eff_ = 4 and 5 μm, the baseline radiative loss is relatively small, so the reduction in transition loss dominates and the optimum appears at *η* = 0.6. For *R*_eff_ = 2 and 3 μm, by contrast, the bend operates in a radiation-dominated regime. Once curvature shaping has mitigated the straight–bend mode mismatch, the residual loss is limited mainly by radiation from the highest-curvature section rather than by the junction condition. The small transition-loss benefit provided by the Euler segment is then offset by the increased radiation caused by the smaller *R*_min_; therefore, no positive *η* further reduces the total loss, and the optimum remains at *η* = 0. This behavior is consistent with partial-Euler analyses, which show that the Euler segment becomes effective only when the baseline radiative loss is sufficiently low for transition-loss reduction to dominate [48].

**Figure 9:**
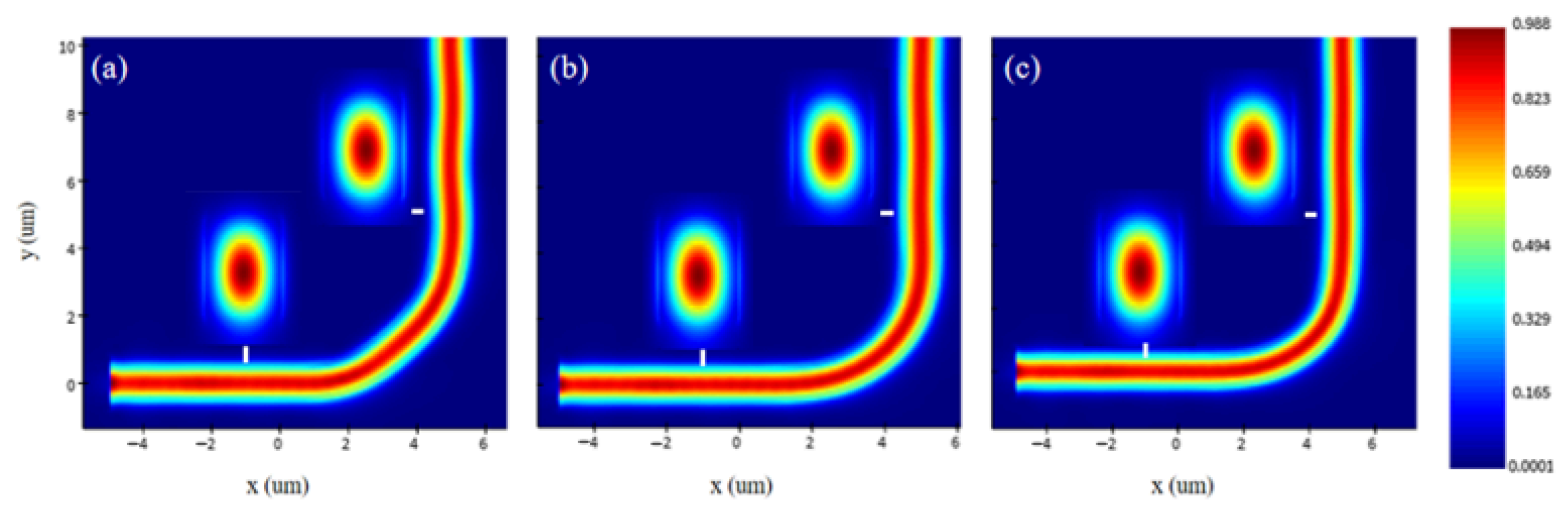
E-field distributions of the Euler–circular hybrid bend with different *η* at a bending radius of 5 μm: (**a**) *η* = 0; (**b**) *η* = 0.6; (**c**) *η* = 0.9.

Figure 9 presents the simulated mode field distributions for different curvature ratios *η*. The embedded insets show transverse E-field profiles extracted at the input cross-section (*x* = −1 μm) and output cross-section (*y* = 5 μm). At *η* = 0 (conventional circular bend, Figure 9a), the field spreads noticeably toward the outer bend side, indicating strong mode mismatch at the straight–bend junction and significant bending-induced radiation leakage, which results in relatively high loss. At *η* = 0.6 (Figure 9b), the mode undergoes a smoother transverse transition, the outer leakage field is substantially suppressed, and the output mode field is more tightly confined, corresponding to the minimum loss. At *η* = 0.9 (Figure 9c), although the curvature transition becomes more continuous, the reduced local minimum bending radius enhances radiation in the rear section of the bend, leading to a slight loss increase relative to the *η* = 0.6 case. These results demonstrate that only with an appropriate curvature ratio can the Euler–circular hybrid bend achieve favorable mode matching and effective radiation suppression simultaneously, thereby realizing optimal low-loss transmission [49]. 

Overall, all three optimization strategies can reduce bending loss, but they act on different aspects of the guided-mode evolution. Their effects can be described in terms of two loss contributions: transition loss at regions of rapid curvature variation or modal discontinuity, which is related to the field overlap between the straight and bent eigenmodes, and distributed radiation loss in the bend, which is associated with the outward displacement of the mode-field centroid and the imaginary part of the bent-mode effective index. The lateral offset mainly reduces transition loss by laterally aligning the input field with the outward-shifted centroid of the bent mode; therefore, its optimum value follows the centroid displacement and becomes larger as the bending radius decreases. Local width reduction instead suppresses the radiative component by lowering the local effective index of the curved section, strengthening lateral confinement, and pulling the mode centroid back toward the waveguide axis, although excessive reduction reintroduces junction mismatch [47]. In contrast, the Euler–circular hybrid bend optimizes the curvature profile rather than introducing an additional offset: by making the curvature *κ*(*s*) continuous at the straight–bend transition, it allows the mode to evolve more adiabatically and reduces intermode coupling [48]. With effective suppression of modal discontinuity and radiative leakage, all the optimized smooth bend structures achieve ultra-low bending loss and stable spectral response, which makes them highly applicable to high-precision photonic devices, including folded resonant cavities and electro-optic interferometers.

### 2.4. Analysis of Pairwise Parameter Interactions

To analyze the coupling interactions among the three smooth bend optimization schemes, pairwise parameter sweeps of Euler proportion *η*, lateral offset Δ*x*, and local width reduction Δ*w* are implemented at *R*_eff_ = 5 μm with consistent simulation parameters. Each parameter pair yields two loss values: the global minimum loss from a single optimization mechanism, and the optimal loss with both mechanisms activated. The difference quantifies the coupling strength of loss-reduction mechanisms, while absolute loss values serve as the primary performance benchmark. The pairwise parameter coupling scan results are summarized in Table 1.

Scan results demonstrate that for all three parameter pairs, the minimum loss with both parameters nonzero is higher than the global optimum, revealing that simple superposition of two optimization strategies cannot further reduce bending loss within the scanned range. Notably, pronounced loss degradation is observed in the two pairs involving the Euler segment. Increasing the Euler proportion *η* smooths the curvature transition at the straight-to-bend junction and reduces transition loss, whereas it simultaneously alters the curvature distribution and bent-mode profile under a fixed effective radius. Accordingly, the individually optimized Δ*x* and Δ*w* for conventional circular bends become incompatible with Euler-modified bend configurations [50]. Direct parameter superposition breaks the inherent balance between interfacial mode matching and radiation suppression, resulting in elevated total loss.

In contrast, the lateral-offset–local-width-reduction pair exhibits a negligible loss difference of only 0.0007 dB/90°, indicating strong physical complementarity between the two strategies. Local width reduction tailors in-bend modal distribution and suppresses outward radiation leakage, while lateral offset compensates residual mode-centroid displacement at the straight–bend interface. Such coordinated modulation enables the dual-parameter optimized loss to closely approach the global minimum. Nevertheless, the optimal state occurs at Δ*x* = 0 nm and Δ*w* = 70 nm, demonstrating that local width reduction dominates mode correction at this radius, and additional lateral offset contributes marginal performance improvement. Overall, the parameter scans confirm that multi-parameter combined optimization improves device performance only when stable modal matching is maintained. Simply increasing the number of optimization parameters does not guarantee lower bending loss.

## 3. Right-Angle Waveguide Bends

A series of previous studies on LNOI photonic devices have clarified the divergent application boundaries between smooth curved bends and right-angle mirror bends [9,11,51]. The three optimized smooth bend schemes proposed above deliver outstanding low-loss transmission and stable spectral performance and are well suited for high-precision photonic devices such as folded resonant cavities and electro-optic interferometers. Benefiting from adiabatic curvature transition, these structures are insensitive to minor sidewall tilt arising from dry etching during fabrication. Nevertheless, their minimum layout size is constrained by the effective bending radius, which severely limits their deployment in ultra-high-density integrated circuits.

In contrast, right-angle (L-shaped) waveguide bends feature compact geometry and negligible radial footprint, which perfectly match the demands of dense integration. Their standardized layout simplifies structural design, wafer fabrication and system-level interconnection, rendering them preferable for grid-style large-scale switch matrices and multi-channel interconnect circuits [46,49,52]. Orthogonal straight optical paths eliminate extra radial occupation and greatly shrink chip area, while total internal reflection at dielectric interfaces enables broadband operation. However, the fabrication of angled reflective facets raises process difficulty, and any deviation from ideal vertical sidewalls will trigger dramatic loss growth, meaning such structures are far more sensitive to imperfect etching profiles. Though their intrinsic transmission loss is notably higher than that of optimized smooth bends, right-angle mirror bends support vertical fiber–chip coupling via grating couplers—a packaging advantage unavailable for smooth curved waveguides [20,23,53].

Considering the unique integration strengths of L-shaped right-angle bends that complement the performance limits of smooth curved structures, this paper conducts a targeted numerical exploration of such bend configurations. Identical to the simulation setup adopted for all smooth bend analyses above, the present work utilizes a z-cut lithium niobate thin-film LNOI platform, with straight waveguide width *w* = 1 μm, waveguide thickness *t* = 0.5 μm and operating wavelength fixed at 1.55 μm. Two loss-mitigation designs, embedded resonant cavities and corner mirror structures, are adopted to suppress steering loss at the straight–bend junction of right-angle bends. The 3D-FDTD method is utilized for full numerical characterization of all device performances.

### 3.1. Embedded Oblique Reflection Resonator Cavity

**Figure 10:**
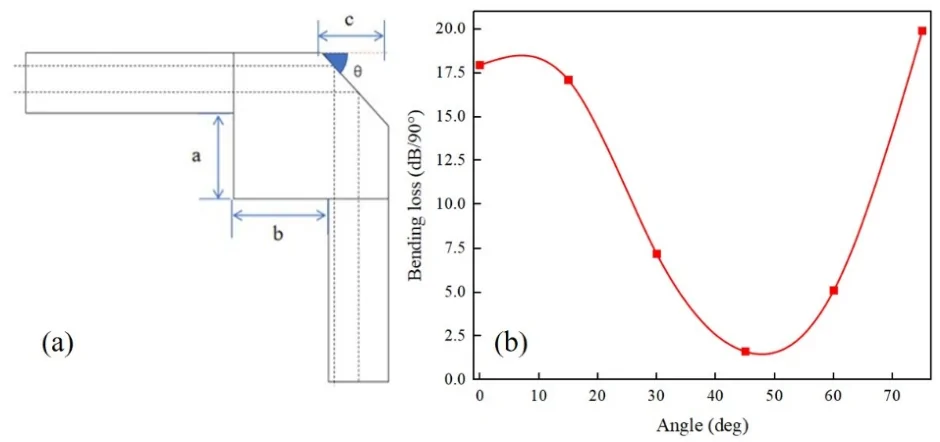
(**a**) Schematic of the embedded oblique reflection resonator cavity; (**b**) relationship between the resonator cavity angle *θ* and bending loss.

To mitigate the severe radiation loss at the corner of a conventional right-angle waveguide bend, an embedded oblique reflection resonator cavity is introduced on the inner side of the 90° bend, as shown in Figure 10a. The geometric parameters are set as *a* = 2 μm, *b* = 2 μm, and *c* = 1.5 μm, with the angle *θ* between the cut facet and the horizontal direction serving as the optimization variable. This structure creates a turning interface based on total internal reflection (TIR) at the corner, redirecting the guided mode from the input straight waveguide at the oblique facet and coupling it into the output waveguide. Consequently, this design achieves lower bending loss than conventional right-angle bends [51,52,54,55].

As depicted in Figure 10b, as *θ* increases from 15° to 75°, the device loss first decreases and then increases, reaching a minimum at *θ* = 45°. For a conventional right-angle bend without the embedded cavity, the loss is 17.96 dB/90°. At the optimal *θ* = 45°, the loss is reduced to 1.62 dB/90°, indicating that the best mode coupling between the reflected field and the output waveguide occurs at this angle. Sharp corners and abrupt turns inherently induce mode distortion and significant optical loss; without a dedicated turning structure, the guided mode suffers substantial radiation leakage at the right-angle bend. The turning behavior can be effectively approximated by the law of reflection and Snell’s law [54]. The 45° mirror is one of the most common configurations for low-loss on-chip turning and coupling, and the mirror angle has been shown to be a key parameter governing bending loss [55]. At *θ* = 45°, incident light is most readily coupled into the output waveguide after a single reflection at the oblique facet. When *θ* deviates from 45°, the reflected light shifts away from the ideal output channel, preventing efficient coupling. The uncoupled energy either undergoes additional reflections within the cavity or adjacent straight waveguides, or it radiates into the cladding, thereby introducing greater excess loss.

**Figure 11:**
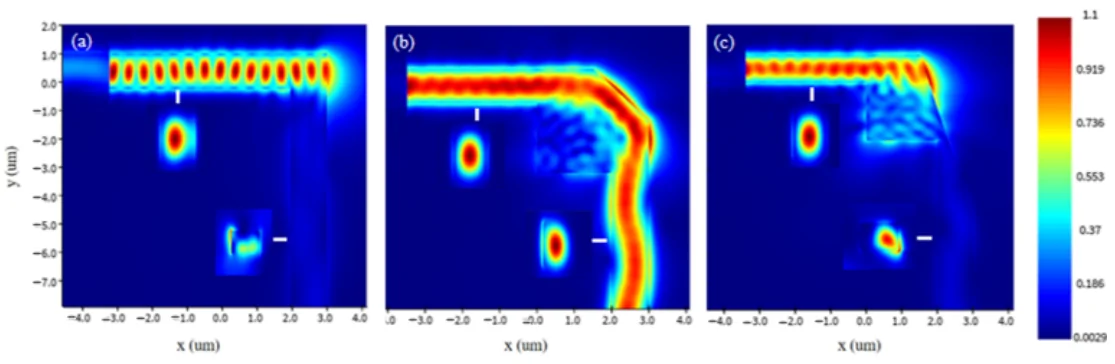
E-field distributions for different resonator cavity angles: (**a**) conventional right-angle bend; (**b**) *θ* = 45°; (**c**) *θ* = 75°.

Figure 11 presents simulated E-field distributions comparing a conventional right-angle bend with resonant-cavity-integrated bends at different tilt angles. The embedded insets show transverse E-field profiles extracted at the input cross-section (*x* = −1.5 μm) and output cross-section (*y* = −5.5 μm). In the absence of a resonant cavity (Figure 11a), the field undergoes severe spreading and distortion at the right-angle bend, accompanied by substantial radiation leakage, resulting in degraded output coupling efficiency and increased propagation loss. In contrast, at *θ* = 45° (Figure 11b), the field is efficiently guided toward the output port upon reflection from the inclined facet. The energy distribution within the bend remains continuous, and the output mode field achieves the highest concentration, indicating optimal mode matching and minimal propagation loss. At *θ* = 75° (Figure 11c), the reflection direction deviates from the output port, with more pronounced field fluctuations and energy diffusion in the corner region, suggesting intensified parasitic reflections and internal leakage. Consequently, the propagation loss increases substantially relative to the *θ* = 45° case. 

### 3.2. Corner Mirror

**Figure 12:**
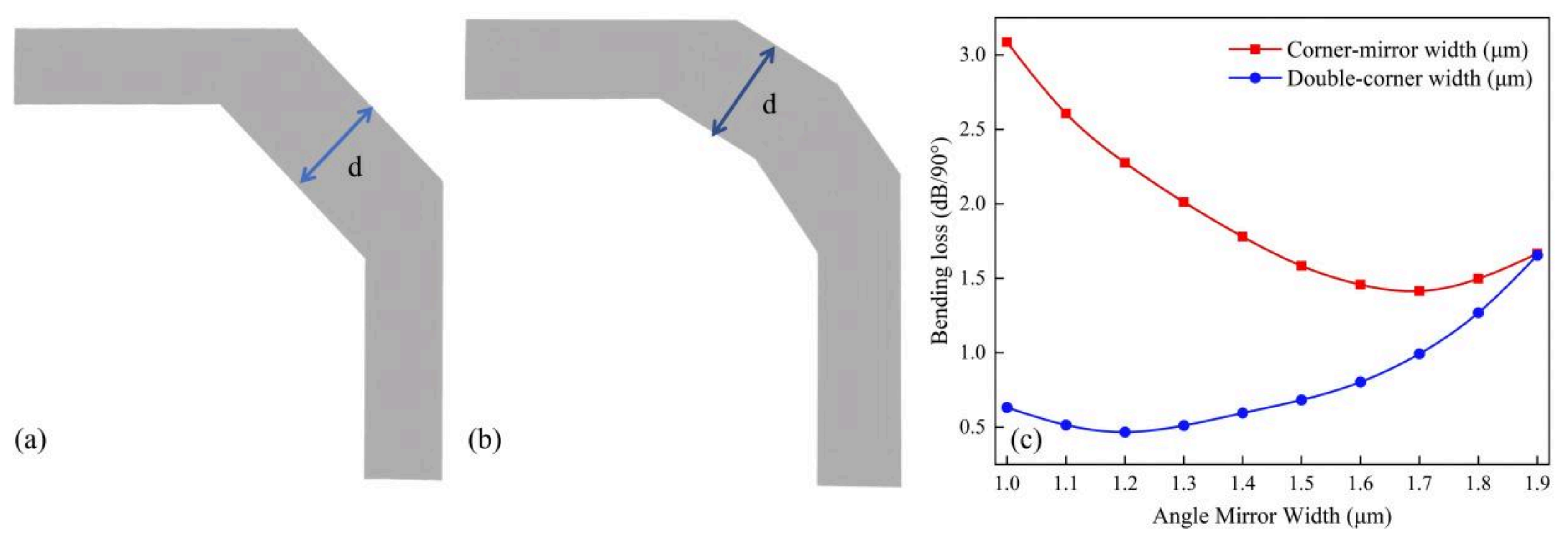
Schematic diagrams of the corner-mirror structures: (**a**) single-corner-mirror configuration; (**b**) double-corner-mirror configuration; (**c**) relationship between corner-mirror width *d* and bending loss.

To reduce the loss of a 90° waveguide bend, a TIR corner-mirror structure is introduced. Two configurations, namely single-corner-mirror and double-corner-mirror schemes, are compared, as illustrated in Figure 12. In the single-mirror design, input light is steered toward the output arm via a single beveled reflective interface. The double-mirror design employs two sequential reflective interfaces for stepwise turning, effectively mitigating the loss associated with a single abrupt deflection. The bending loss is further optimized by varying the mirror width *d*.

The bending loss of the right-angle waveguide is 17.96 dB/90°. As shown in Figure 12c, as the mirror width d increases, the losses of both single- and double-corner-mirror structures first decrease and then increase. For the single-mirror structure, the minimum loss is 1.41 dB/90° at *d* = 1.70 μm, a reduction of 16.55 dB/90° compared with the right-angle waveguide. For the double-mirror structure, the width-optimized minimum is 0.47 dB/90° at *d* = 1.20 μm, a reduction of 17.49 dB/90°, and the loss remains low for d between 1.10 and 1.35 μm before increasing at larger widths. The inter-mirror angle *θ* was also investigated over 0–90°; the loss first decreases and then increases with *θ*, reaching a minimum of 0.47 dB/90° at *θ* = 60°, which is 17.49 dB/90° lower than that of the right-angle waveguide. Overall, the optimized double-corner-mirror configuration shows lower loss than the single-mirror design within the investigated parameter range.

**Figure 13:**
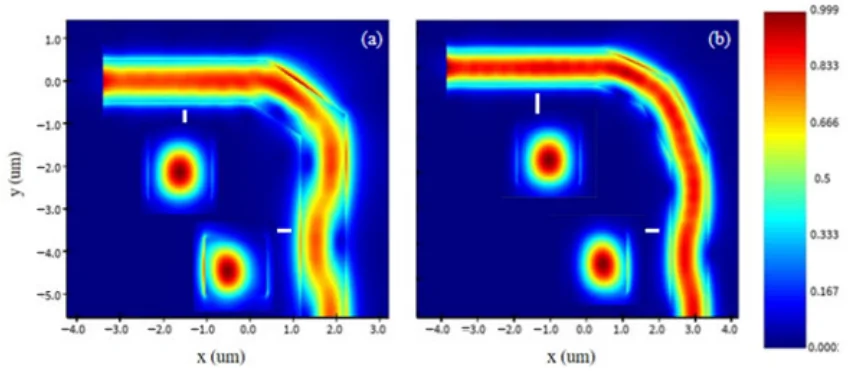
Electric field distributions for (**a**) single-corner-mirror and (**b**) double-corner-mirror structures.

As shown in Figure 13, the embedded insets show transverse E-field profiles extracted at the input cross-section (x = −1.5 μm) and output cross-section (y = −3.5 μm). The single-corner-mirror structure exhibits noticeable local field distortion and leakage near the reflective interface and the inner corner, along with uneven reflected energy before entering the output port. In contrast, the double-corner-mirror structure provides a more continuous field distribution in the corner region, reduced external leakage, and a smoother, more concentrated field at the output waveguide, indicating lower total loss and improved energy transmission through the corner. A sharp right-angle bend inherently causes significant mode variation and radiation loss. Introducing a reflective interface redirects light at the mirror surface, preventing direct radiation into the outer cladding and thereby reducing loss [53]. An excessively small mirror width offers insufficient reflection for effective turning, whereas an overly large width degrades mode matching between the reflected field and the output port, increasing scattering and leakage. The double-corner-mirror structure shows lower loss than its single-mirror counterpart by decomposing a single abrupt turn into two gradual reflection processes, improving bending transmission with a limited increase in footprint. Consequently, it mitigates the localized high field intensity near the inner corner and the outward radiation observed in the single-mirror case, enabling a smoother turning process and ultimately lower bending loss [56]. 

**Figure 14:**
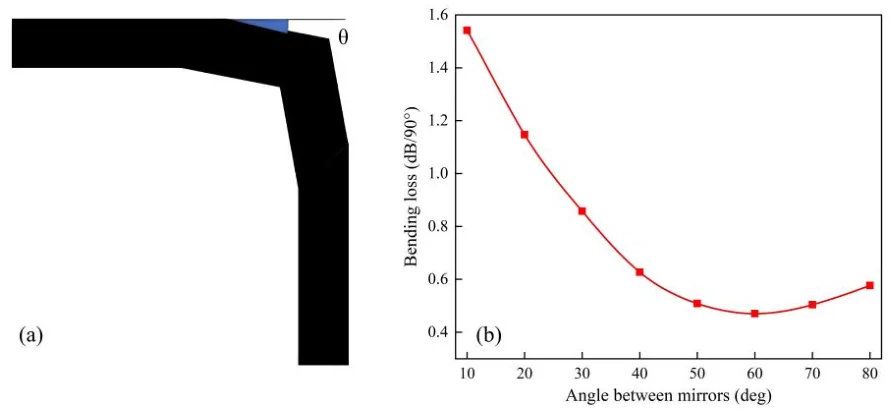
(**a**) Schematic diagram of the included angle *θ* in the double-corner-mirror structure; (**b**) relationship between the included angle *θ* and bending loss.

The angular configuration of the double-corner-mirror structure is further investigated in Figure 14a. The structure consists of two sequentially connected beveled reflective interfaces with an included angle *θ*, enabling a 90° turn via successive TIR. As shown in Figure 14b, as *θ* increases from 0° to about 60°, the bending loss decreases from approximately 1.93 dB/90° to a minimum of 0.47 dB/90° and then rises slightly for larger angles, indicating that an included angle near 60° yields the best turning transmission performance. Loss in a 90° bend mainly originates from corner radiation leakage and post-reflection mode mismatch. Corner mirrors mitigate radiation loss by redirecting the propagation direction. A larger *θ* provides a smoother overall reflection path, gradually reduces radiation leakage at the mirror junctions, and improves mode matching at the output port, minimizing the total loss at an included angle of about 60°, beyond which the loss rises slightly as output-coupling mismatch grows.

Among the three right-angle turning structures compared here, namely the embedded resonant cavity, the single-corner mirror, and the double-corner mirror, the double-corner mirror gives the lowest loss within the investigated parameter range, whereas the resonant-cavity design shows the poorest transmission. This hierarchy can be attributed to their different turning mechanisms. A corner mirror redirects the guided mode through total internal reflection at a dielectric–air interface, which is broadband and can be nearly lossless for an ideal reflecting facet; similar total-internal-reflection corner bends have been reported to maintain excess loss below 1 dB over the telecommunication band [51]. By contrast, the embedded cavity turns light by field buildup and re-coupling in a finite-Q resonator, making the response wavelength- and phase-selective and causing uncoupled power to remain in the cavity or leak into the cladding. This mechanism is consistent with the higher cavity loss obtained here (1.62 dB/90°) compared with the optimized double mirror (0.47 dB/90°). The double-corner mirror further improves upon the single mirror because the 90° turn is divided into two milder reflections, keeping each reflection closer to the efficient total-internal-reflection range, reducing diffraction leakage, and improving output mode matching with only a small footprint penalty. Therefore, the double-corner mirror shows lower simulated loss than both the single-corner mirror and the resonant-cavity bend, in agreement with earlier high-index-contrast right-angle-bend studies [56].

### 3.3. Broadband Wavelength-Dependent Performance

**Figure 15:**
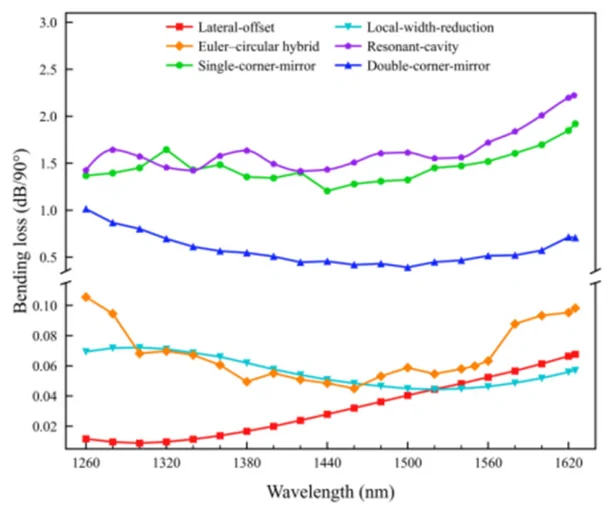
Simulated bending losses of the six optimized 90° waveguide bends across the 1260–1625 nm spectral range.

To evaluate the applicability of the six optimized bend structures across optical telecommunications bands, the 90° bend loss was scanned from 1260 to 1625 nm under fixed geometrical parameters, waveguide cross-section, boundary conditions, and monitor settings. Figure 15 shows that the three smooth bends retain low and comparatively flat losses within the C band. From 1540 to 1560 nm, the local-width-reduction, lateral-offset, and Euler–circular hybrid bends vary from 0.045 to 0.046 dB/90°, from 0.048 to 0.053 dB/90°, and from 0.058 to 0.063 dB/90°, respectively. Among the right-angle structures, the double-corner-mirror loss increases from 0.466 to 0.514 dB/90° and remains the lowest, whereas the resonant-cavity loss increases from 1.541 to 1.735 dB/90° and exhibits the strongest wavelength dependence.

The distinct spectral behaviors of the proposed bends are mainly governed by the wavelength dependence of modal confinement, transverse field distribution, and straight-to-bend mode matching under fixed waveguide geometries. As the operating wavelength increases, the guided mode extends further into the cladding layer, enlarging the field proportion near the outer bend and exacerbating radiation loss and interface mode mismatch, which causes the general upward loss trend at longer wavelengths [57]. For the three smooth bends, each structure mitigates wavelength-induced degradation through different mechanisms. The local-width-reduction design optimizes the modal confinement and field distribution inside the bend, effectively balancing radiation loss and mode mismatch to achieve superior spectral flatness across the C band. The fixed lateral-offset structure effectively compensates for the outward displacement of bend modes, whereas its compensation capability weakens at longer wavelengths, resulting in gradual loss deterioration. The Euler–circular hybrid bend eliminates abrupt curvature transitions to reduce sudden mode disturbance; nevertheless, transition loss and distributed bending radiation respond differently to wavelength variation, thereby producing a mild non-monotonic spectral characteristic. For total-internal-reflection mirror bends, double-angle reflection improves field reconstruction and output coupling efficiency, enabling the double-corner mirror to maintain much lower loss than the single-corner structure, while both mirror designs exhibit comparable spectral fluctuation ranges. In contrast, the resonant-cavity bend possesses strong wavelength sensitivity, since its performance relies heavily on intracavity phase accumulation and resonant field enhancement, which are inherently frequency-selective. Overall, the optimized smooth bends enable stable, low-loss broadband routing for C-band high-speed optical communications and wavelength-division multiplexing systems. It is worth noting that these passive spectral characteristics describe only the transmission performance of waveguide components and cannot directly represent the electro-optic modulation bandwidth and data rate of fully integrated active photonic systems.

### 3.4. Fabrication Tolerance Analysis

Fabricated LNOI waveguides deviate from the ideal vertical-sidewall geometry under the combined influence of lithography and dry etching imperfections, as well as pre-fabrication processes such as wafer bonding and polishing, which collectively produce four types of dimensional deviations: waveguide width, etch depth, sidewall angle and LN film thickness. Deviations in waveguide width, etch depth and sidewall angle are introduced by etching, while inconsistencies in film thickness originate from the early substrate preparation stage. During thin-film lithium niobate dry etching, limited mask selectivity, physical ion bombardment and material redeposition jointly shape trapezoidal ridge cross-sections with inclined sidewalls [9,24,30]. In this section, continuous multi-parameter sweep simulations are carried out to quantitatively characterize the fabrication tolerance of all six optimized 90° bend configurations.

A single-variable simulation method is adopted in this work. The geometric parameters swept sequentially include waveguide width (900–1100 nm), LN film thickness (450–550 nm), etch depth (400–500 nm), and sidewall angle (60–75°). Only one parameter is varied at a time, while the overall geometry of the bending structure, material dispersion model, incident light source, field monitor settings, and all boundary conditions remain unchanged. The nominal reference structure has an effective bending radius *R*_eff_ = 5 μm, waveguide width of 1 μm, LN film thickness of 500 nm, full etch depth of 500 nm, and perfectly vertical sidewalls.

**Figure 16:**
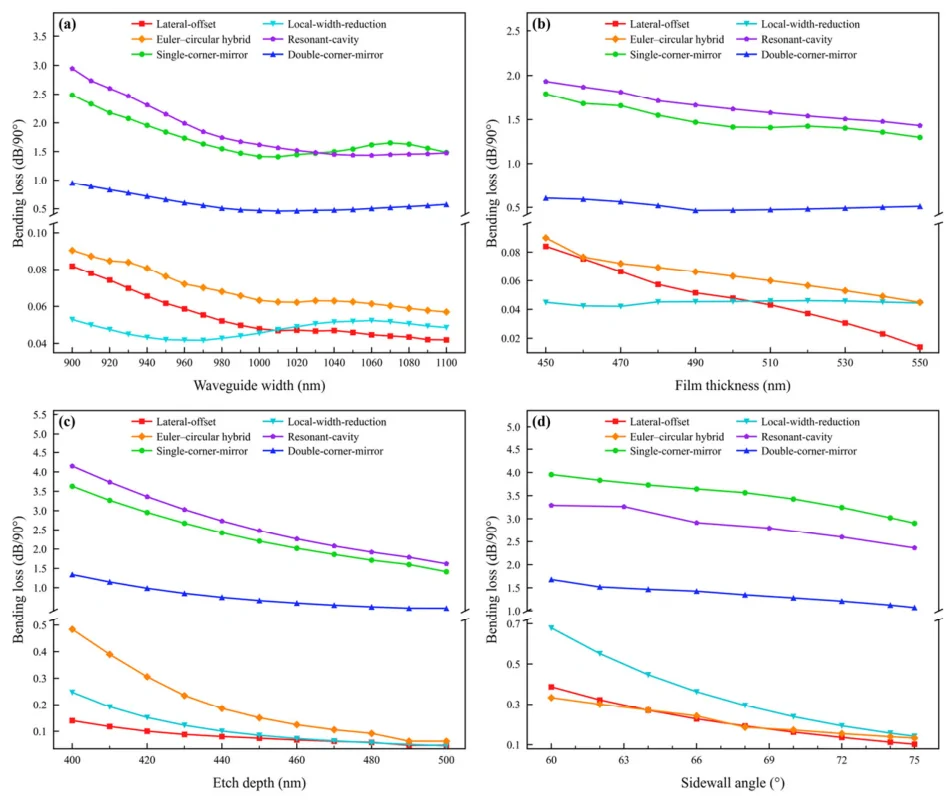
Simulated 90° bending loss of the six optimized waveguide bends as a function of fabrication parameters: (**a**) waveguide width (900–1100 nm); (**b**) LN film thickness (450–550 nm); (**c**) etch depth (400–500 nm); (**d**) sidewall angle (60–75°).

Figure 16 presents the loss variations of the six bending structures under different fabrication deviations, revealing distinct responses to the four parameters. As shown in Figure 16a, reducing the waveguide width from 1000 nm significantly increases the loss of lateral-offset and Euler–circular hybrid bends, whereas the local-width-reduction bend shows excellent stability, with loss fluctuating only within 0.042–0.053 dB/90°. Right-angle bends are more sensitive to width variations due to modified modal confinement and mode mismatch at the straight–bend interface; in addition, sharp-turn structures suffer from diffraction and field distortion in the bending region, further reducing transmission performance. As illustrated in Figure 16b, increasing the LN film thickness strengthens vertical optical confinement and suppresses sidewall scattering loss, thus lowering loss for most structures. The local-width-reduction bend remains highly stable at 0.042–0.046 dB/90° over the thickness range, and the double-corner-mirror bend reaches minimum loss near the nominal 500 nm thickness, corresponding to optimal field reconstruction after double reflections and efficient output mode matching [56]. As shown in Figure 16c, insufficient etching leaves a thick residual LN slab, weakening lateral modal confinement and exciting parasitic leaky slab modes. Strong coupling between leaky modes and the fundamental guided mode causes a sharp rise in propagation loss, especially for right-angle structures. As observed in Figure 16d, loss of all structures decreases continuously as the sidewall angle approaches 90° and the waveguide cross-section approaches the ideal vertical profile. Smooth bends inherently show low insertion loss and stable performance, while resonant-cavity and single-corner-mirror bends exhibit significantly increased loss under large sidewall inclination. In contrast, the double-corner-mirror structure maintains the lowest loss among all right-angle designs within the investigated angle range.

Overall, the fabrication tolerance analysis reveals that the structural sensitivity to process deviations is dominated by the waveguide bending mechanism. Variations in waveguide width and film thickness exert marginal impacts on smooth bends, among which the local-width-reduction configuration achieves the minimum loss fluctuation under both width and thickness perturbations. Insufficient etching and inclined sidewalls induce substantial loss elevation, particularly for sharp-turn structures. Compared with other right-angle bending schemes, the double-corner-mirror structure consistently delivers the lowest propagation loss and the slightest performance variation under process deviations.

### 3.5. Comparison with Reported Experimental Data

To verify the physical validity of the simulated bending loss values under identical miniaturization standards, a comprehensive comparison is performed against a range of experimentally characterized thin-film lithium niobate (TFLN/LNOI) waveguide bends with micrometer-scale radii and comparable compact footprints. Free-form hybrid waveguides combining TFLN and chalcogenide glass were fabricated and measured by Wan et al.; their 90° bend with an effective radius of 10 μm achieves a single-turn loss of approximately 0.25 dB [36]. Inverse-designed 90° bends were realized on Z-cut LNOI substrates by Shang et al. The structure with a 6 μm curvature radius delivers an average insertion loss of ~0.41 dB across the 1500–1600 nm telecommunication band [37]. Additional published experimental demonstrations with similar compact dimensions further support the comparison framework. 10 μm cubic Bézier bends were integrated into monolithically fabricated Z-cut TFLN spiral modulators for on-chip light routing by Ghoname et al. [38]. Polarization devices constructed with width-tapered 180° Euler bends featuring a minimum radius of 10 μm were reported by Liu et al., and the full device maintains an excess loss below 1.5 dB within the 1480–1578 nm operating window [39]. An inverse-designed X-cut TFLN bend with a footprint of 18.5 × 18.5 μm^2^ was experimentally characterized by Lyu et al., with a single-bend loss of roughly 1.23 dB extracted at 1550 nm [40].

Numerical simulations reveal that the three optimized smooth bends proposed in this work exhibit bending losses ranging from 0.050 to 0.060 dB per 90° turn at an equivalent radius *R*_eff_ = 5 μm. For reference, conventional circular bends under the same dimensional conditions reach a loss of 0.120 dB/90°. The 5 μm compact bend proposed herein, the 6–10 μm curved structures reported in the above references, and the 18.5 × 18.5 μm^2^ inverse-designed bend all fall into the category of micrometer-scale interconnects suitable for high-density photonic integration. Refs. [36,37] act as the most suitable quantitative benchmarks, as both report measured single-bend losses below 0.5 dB, with numerical deviations from the simulated results smaller than one order of magnitude. Supplementary experimental evidence for miniaturized LNOI routing components is provided by Refs. [38,39,40]. It should be noted that the waveguide designs from cited works differ from the present scheme in material stacks, etched cross-section profiles and intended device functions. This cross-literature comparison is therefore only used to confirm that the simulated loss magnitudes are physically reasonable rather than to draw direct performance comparisons between separate photonic platforms.

## 4. Conclusions

This study systematically compares six 90° LNOI routing structures within a consistent 3D-FDTD framework and relates their geometries to the dominant mechanisms of bending loss. In smooth bends, the lateral offset improves mode matching at the straight-to-bend junction, local width reduction modulates modal confinement in the curved section and thereby suppresses outward radiation, and the Euler–circular hybrid curve reduces junction discontinuities through a continuous curvature transition. In right-angle bends, the double-corner-mirror configuration divides one abrupt turn into two reflections, improving field reconstruction and output-mode matching. At an effective radius of 5 μm, the three optimized smooth bends exhibit losses of 0.046–0.063 dB/90° compared with 0.120 dB/90° for the conventional circular bend, while the optimized double-corner-mirror bend yields 0.467 dB/90°, the lowest value among the investigated right-angle structures. Pairwise parameter scans also reveal non-additive coupling among the Euler proportion, lateral offset, and local width reduction.

Wavelength sweeps over 1260–1625 nm show that all three smooth bends maintain low, comparatively flat loss across the C band, with the local-width-reduction design providing the best spectral flatness, whereas the double-corner-mirror bend has the lowest wavelength-dependent loss among the right-angle configurations. Fabrication-tolerance analysis identifies reduced etch depth and increased sidewall inclination as the principal sources of loss degradation; the local-width-reduction bend remains comparatively stable against variations in waveguide width and LN film thickness, and the double-corner-mirror bend retains the lowest absolute loss among the right-angle designs. Tests of mesh refinement, PML boundary distance, and an independent eigenmode expansion model limit numerical discrepancies to below 0.010 dB, supporting the reliability of the reported loss trends. These findings clarify the relationships among bend geometry, loss-reduction mechanism, parameter coupling, spectral response, and fabrication sensitivity in compact LNOI routing structures and support the selection of compact, low-loss bend geometries for C-band LNOI passive routing.

## Figures and Tables

**Table 1:** Results of pairwise parameter coupling scans at *R*_eff_ = 5 μm.

Effective Radius	Parameter Pair	Global Optimum	Global Minimum Loss (dB/90°)	Best Interior Point	Best Interior Loss (dB/90°)	Loss Difference (dB/90°)
5 μm	Euler–lateral offset	*η* = 0; Δ*x* = 50 nm	0.0487	*η* = 0.05; Δ*x* = 40 nm	0.0635	+0.0148
5 μm	Euler–local width reduction	*η* = 0; Δ*w* = 70 nm	0.0466	*η* = 0.05; Δ*w* = 70 nm	0.0576	+0.0110
5 μm	Lateral offset–local width reduction	Δ*x* = 0 nm; Δ*w* = 70 nm	0.0466	Δ*x* = 40 nm; Δ*w* = 10 nm	0.0473	+0.0007

## Data Availability

All results supporting the conclusions of this article were obtained from numerical simulations. Key simulation parameters are fully presented within the manuscript. Raw simulation files are not deposited in public repositories, but the corresponding author will provide relevant raw data upon reasonable request.

## Appendix A

### Appendix A.1. Numerical Simulation Details

This appendix presents the comprehensive numerical parameter configurations adopted in the finite-difference time-domain (FDTD) simulations, which serve as the fundamental data basis for the device performance analysis and numerical results demonstrated in the main manuscript. All key parameters applied in the FDTD numerical calculations are systematically summarized in Table A1.

**Table A1:** Summary of numerical simulation parameters used in the FDTD calculations.

Category	Parameter	Setting Used in This Work
Numerical solver	Solver	Three-dimensional finite-difference time-domain solver (Ansys Lumerical FDTD, 2025 R2)
Material model	LN guiding layer	Z-cut lithium niobate modeled as a uniaxial anisotropic crystal
Material model	LN refractive indices	Ordinary and extraordinary refractive indices set to *n*_o_ = 2.211 and *n*_e_ = 2.138 at 1.55 μm
Material model	Cladding	Silicon dioxide cladding with refractive index *n* = 1.444
Waveguide geometry	Core dimensions	Film thickness *t* = 0.5 μm, and straight-waveguide width *w* = 1 μm
Excitation	Source mode	TE mode launched along the +x direction
Simulation region	Computational window	Complete bend plus sufficiently long straight input and output access waveguides
Boundary condition	Outer boundaries	Perfectly matched layers applied on all six simulation boundaries
Mesh	Mesh type	Conformal nonuniform mesh; the override region covered the waveguide and inclined sidewalls
Mesh	Mesh resolution	The main parameter sweeps used in-plane steps Δ*x* = Δ*y* = 10 nm. For mesh-convergence verification, the optimized structures were additionally evaluated using Δ*x* = Δ*y* = 10, 7, and 5 nm; the out-of-plane step was kept at Δ*z* = 20 nm
Mesh	Discretization	Refined mesh discretized into 212 × 270 × 56 Yee cells; mesh density progressively increased
Monitors	Power and mode analysis	Frequency-domain power monitors at the input and output waveguides, together with a mode expansion analysis monitor
Calculated quantities	Transmission and loss	Transmittance *T* = *P*_out_/*P*_in_; bend loss Loss = −10 log_10_(*T*), reported in dB/90°
Modeling choice	LN film thickness	A 500 nm LN film thickness was adopted to satisfy the single-mode condition and maintain vertical optical-field confinement

### Appendix A.2. Numerical Convergence and Independent-Model Validation

Because some of the optimized bend losses are only approximately 0.0500–0.0600 dB/90°, discretization error, computational-domain truncation, and model-specific numerical bias could affect small loss differences among the structures. We therefore performed in-plane mesh refinement, PML-boundary-distance checks, and independent-model cross-validation. In each convergence test, only one numerical control parameter was varied, while all other simulation settings were held fixed.

#### Appendix A.2.1. In-Plane Mesh Convergence

Mesh-convergence tests were performed for all six optimized structures. Among them, the local-width-reduction bend combines a low bend loss with a small geometric variation scale and was therefore selected for a more stringent grid-refinement test. With the device geometry, material parameters, source, monitors, out-of-plane mesh, and boundary conditions held unchanged, the in-plane mesh steps Δ*x* = Δ*y* were set successively to 10, 7, and 5 nm. All losses were calculated as −10 log_10_ |*S*_21_|^2^. At in-plane mesh steps of 10, 7, and 5 nm, the local-width-reduction bend losses were 0.0553, 0.0461, and 0.0468 dB/90°, respectively. Compared with the 5 nm result, the 10 and 7 nm results differed by 0.0085 and 0.0007 dB, respectively.

The total difference between the 10 and 5 nm results was 0.0085 dB, corresponding to a linear-transmittance difference of approximately 0.1955%, and remained below the 0.0100 dB loss scale considered in this work. The 7 and 5 nm results were nearly identical, and the other five optimized structures showed the same convergence trend. The 10 nm mesh used in the main parameter sweeps therefore resolves the loss trend, while the finer-mesh calculations verify the reported optimized losses.

#### Appendix A.2.2. PML-Boundary-Distance Convergence

The local-width-reduction bend was also selected for the PML-distance check because its low bend loss makes it sensitive to small numerical errors arising from boundary reflections. The minimum distance between the waveguide core and the PML was varied while the local-width-reduction geometry, mesh, material parameters, source, monitors, PML type, and number of PML layers were held fixed. For minimum core-to-PML distances of 1.25, 1.50, and 2.00 μm, the calculated 90° bend losses were 0.0446, 0.0446, and 0.0444 dB/90°, respectively.

The maximum spread among the three PML-distance results was 0.0002 dB/90°, which remained substantially below 0.0100 dB/90°. The loss remained essentially unchanged between 1.25 and 1.50 μm and decreased only slightly at 2.00 μm. Thus, within the tested range, the PML position does not alter the low-loss conclusion for the local-width-reduction bend.

#### Appendix A.2.3. Independent-Model Cross-Validation

To further exclude systematic bias associated with the FDTD discretization or a single numerical model, all six optimized structures were independently modeled using the same operating wavelength, polarization, material parameters, and device geometry. The EME validation results were expressed in terms of the fundamental-mode transmission coefficient *S*_21_ and converted to loss using −10 log_10_ |*S*_21_|^2^. The FDTD–EME cross-validation results are summarized in Table A2.

**Table A2:** FDTD–EME cross-validation of the calculated 90° bend losses.

Structure	FDTD Loss (dB/90°)	EME Loss (dB/90°)
Lateral-offset	0.0480	0.0490
Local-width-reduction	0.0461	0.0456
Euler–circular hybrid	0.0632	0.0634
Resonant-cavity	1.6190	1.6200
Single-corner-mirror	1.4146	1.4150
Double-corner-mirror	0.4670	0.4660

The FDTD and EME losses were in close agreement for all six optimized structures. For the lateral-offset, resonant-cavity, single-corner-mirror, and double-corner-mirror bends, the absolute differences between the two calculations were approximately 0.0010 dB. For the local-width-reduction and Euler–circular hybrid bends, the absolute differences were 0.0005 and 0.0002 dB, respectively. Comparable agreement was therefore obtained across structures with different turning mechanisms and loss levels.

All FDTD–EME differences were substantially below 0.0100 dB, amounting to approximately one tenth or less of the loss scale of interest in this work. This agreement across independent models demonstrates that the low-loss FDTD results are reproducible and do not depend on a single numerical solver. Taken together, the mesh-refinement, PML-distance, and independent-model results show that all identified numerical variations were below 0.0100 dB.
